# The Mechanisms of Mindfulness in the Treatment of Mental Illness and Addiction

**DOI:** 10.1007/s11469-016-9653-7

**Published:** 2016-05-13

**Authors:** Edo Shonin, William Van Gordon

**Affiliations:** 1 Division of Psychology, Nottingham Trent University, Chaucer Building, Burton Street, Nottingham, NG1 4BU UK; 2 Awake to Wisdom Centre for Meditation and Mindfulness Research, Nottingham, UK

**Keywords:** Mindfulness, Mechanisms of action, Second-Generation mindfulness-based, Interventions, Meditation, Addiction treatment, Mental illness

## Abstract

Consistent with its growing popularity amongst the general public and medical community, throughout recent decades there have been increasing attempts to understand the mechanisms that underlie therapeutic improvement in individuals receiving mindfulness training. The current paper draws upon findings from various remits of scientific enquiry and summarises key evidence-based mechanisms of mindfulness that have been proposed in the academic literature to date. Empirical findings indicate that mindfulness targets biological, psychological, social, and spiritual psychopathology determinants. Furthermore, the mechanistic pathways exploited by mindfulness are likely to vary according to factors such as (i) the type of mindfulness-based intervention that is administered (e.g., first- or second-generation mindfulness-based interventions), (ii) the specific clinical disorder that is being targeted, (iii) the educational, social, and spiritual history of the participant, and (iv) the extent to which the mindfulness instructor truly embodies the principles of mindful living. It is hoped that the mechanisms of mindfulness discussed in this paper will contribute to the formulation of a more complete picture that can be tested and expanded upon during future scientific enquiry.

Mindfulness is a 2600-year-old meditation technique that derives from Buddhist practice. Although contemplative techniques similar to mindfulness feature in many of the world’s religious and spiritual traditions, the type of mindfulness employed in mental health settings is largely based on the Buddhist model. A number of discourses or *suttas* (Pali; Sanskrit: *sutras*) in the Buddhist canonical literature are specifically devoted to the practice of mindfulness, of which the principal ones are the *ānāpānasati sutta*, *satipaṭṭhāna sutta*, *mahasatipaṭṭhāna sutta*, and *kāyagatāsati sutta*. Consequently, we would argue that ‘mindfulness’ – that is a direct translation of the Buddhist Pali term ‘*sati*’ – assumes a more pivotal role in Buddhism compared to other religions.

In recent years, different delineations of mindfulness have been proposed that largely reflect the preference to either distance or align contemporary mindfulness-based interventions (MBIs) with their Buddhist and spiritual roots. An example of the former type of delineation was proposed by Kabat-Zinn who asserts that mindfulness is the process of “*paying attention in a particular way*: *on purpose*, *in the present moment*, *and non-judgmentally*” (1994, p.4). An example of the latter was proposed by Shonin and Van Gordon who define mindfulness as the “*process of engaging a full*, *direct*, *and active awareness of experienced phenomena that is* (*i*) *spiritual in aspect*, *and* (*ii*) *maintained from one moment to the next*” (2015, p. 900). Kabat-Zinn’s delineation is typically embodied by first-generation mindfulness-based interventions such as Mindfulness-Based Stress Reduction and Mindfulness-Based Cognitive Therapy, whereas the definition proposed by Shonin and Van Gordon – that specifically refers to mindfulness as a ‘spiritual’ practice – is embodied by second-generation MBIs such as Meditation Awareness Training.

Although mindfulness does not necessarily require the use of breath awareness, in mental health settings participants of MBIs are typically instructed to use observance of breathing as an attentional anchor. Focussing attention on the natural flow of the in and out breath is understood to arrest ruminative thinking patterns and thus permit a degree of meditative concentration to arise (Harvey, 2015). Eventually, participants may be able to maintain awareness of the present moment without using an attentional anchor, but this is a reasonably advanced form of meditative practice. Furthermore, even in the event an individual is able to observe the present moment without relying on an attentional referent such as observing the breath, it could be argued they are simply adopting the ‘present moment’ as their attentional anchor and are thus still relying on a meditative prop.

To differing degrees, mindfulness is advocated by the United Kingdom’s National Institute for Health and Care Excellence (NICE), American Psychiatric Association (APA), and Royal Australian and New Zealand College of Psychiatrists (RANZCP) for the treatment in adult populations of recurrent depression (NICE and APA) and binge eating disorder (RANZCP). Based on the above recommendations and emerging empirical evidence, the United Kingdom’s Mental Health Foundation recently called for mindfulness to be made more readily available through the National Health Service (Van Gordon et al., 2016a). The growing popularity of mindfulness in mental health settings is likewise indicated by the increase in mindfulness publication output. For example, papers concerning mindfulness published in academic journals have undergone a tenfold increase in the last ten years, with over 700 such papers published during 2015 (American Mindfulness Research Association, 2016).

Consistent with its growing popularity amongst the general public and medical community, throughout recent decades there have been increasing attempts by scientists to understand the mechanisms that underlie therapeutic improvement in individuals receiving mindfulness training. However, coming to a definitive conclusion regarding the precise mechanisms of action that underlie the biological, psychological, and spiritual changes induced by mindfulness is problematic because MBIs typically include a range of therapeutic techniques (e.g., mindfulness, group discussion, silent contemplation, one-to-one discussion, yoga exercises, psycho-education, etc.). Consequently, as with many other non-pharmacological treatments, proposals in terms of how mindfulness improves mental health or aspects of human behaviour are generally regarded to constitute just one component in a multi-mechanistic process. This paper draws upon findings from various remits of scientific enquiry and summarises what the present authors consider to be ten noteworthy and evidence-based mechanisms of mindfulness that have been proposed in the academic literature to date.

1. ***Structural Brain Changes***: Neuropsychological imaging studies have demonstrated that mindfulness can induce neuroplastic changes in various areas of the brain (including the anterior cingulate cortex, insula, default mode network structures, left hippocampus, temporo-parietal junction, and fronto-limbic network; Holzel et al., 2011a). The structural brain changes linked to mindfulness (and related forms of meditation) are typically associated with (i) increased learning and memory capacity (Holzel et al., 2011b), (ii) improved self-regulatory efficacy (i.e., an individual’s ability to regulate and remain in control of their choices, feelings, and behaviours; Luders et al., 2009; Tang et al., 2012), and (iii) greater interoceptive awareness (i.e., increased sensitivity to sensations that originate in the body as well as associated subjective awareness of the physical self as a sentient being; Lazar et al., 2005).
2. ***Reduced Autonomic Arousal***: Mindfulness and related meditative techniques have been shown to increase output in the vagus nerve – a primary cranial nerve responsible for regulating heart and breathing rate (Telles et al., 2013). In turn, reduced autonomic arousal is associated with increased relaxation response, including across both somatic and psychological relaxation indices (Khanna & Greeson, 2013). These findings are consistent with Buddhist teachings such as the aforementioned *ānāpānasati sutta* that outlines 16 breathing exercises specifically intended to induce bodily and mental stillness in order to foster (i) blissful affective states and (ii) spiritual insight.
3. ***Perceptual Shift***: Practising mindfulness is believed to create a perceptual shift in the way individuals respond and relate to thoughts, feelings, and sensory stimuli (e.g., sounds, sights, smells, pain, etc.; Jerath et al., 2012). This greater perceptual distance is understood to encourage the objectification of distressing psychological or painful bodily experiences (Ludwig & Kabat-Zinn, 2008). Likewise, perceptually ‘stepping back’ and observing the mind and body as they interact with the present moment can reduce inclination towards anger and help individuals regard psychological and sensory processes as ‘passing experiences’ (Shonin et al., 2015).
4. ***Increase in Spirituality***: Mindfulness, particularly when practiced according to the second-generation model, can increase spirituality that helps to buffer against feelings of loneliness or being overwhelmed by life adversities (Van Gordon et al., 2016b). This growth in spiritual awareness is understood to help broaden an individual’s life perspective and cause them to re-evaluate their life priorities. Second-generation MBIs have also led to participants ‘glimpsing’ what might be described as profound or nuance spiritual truths such as non-self and emptiness (i.e., the notion that the self – and indeed all phenomena – are composite and devoid of intrinsic existence; Van Gordon et al., 2016b). A further example of growth in spirituality following receipt of an MBI is a greater awareness of death, including the fact that it is an inevitable occurrence. Increased death awareness is understood to facilitate the earlier-onset of the recovery and restorative phases of the grieving process, and to build resilience to trauma (Cacciatore & Rubin, 2015; Kumar, 2005; Wada & Park, 2009).
5. ***Greater Situational Awareness***: Mindfulness can help individuals better understand and connect with the physical and social environment in which they find themselves. More specifically, it can induce what has been termed the *phenomena feedback effect* that involves the mindfulness practitioner entering into a form of effortless communication with the present moment (Shonin & Van Gordon, 2015). This greater situational awareness is understood to improve (amongst other things) decision-making competency, job performance, and the ability to anticipate how a particular situation might unfold (Shonin & Van Gordon, 2015).
6. ***Values Clarification*:** Values clarification is the recognition by individuals of what they truly value and find meaningful in life (Shapiro et al., 2006). Values clarification has been shown to partially mediate the relationship between mindfulness and psychological symptom reduction, suggesting that mindful awareness helps to clarify purpose in life that subsequently leads to improved mental health outcomes (Carmody et al., 2009). There are various explanations as to why mindfulness causes a clarification or reappraisal of life values but a plausible one is that the mindfulness practitioner begins to appreciate more fully that life can only unfold and be experienced in the present moment, and that fantasising about the future or ruminating over the past is ultimately a futile and fatiguing process.
7. ***Increase in Self-Awareness***: Mindfulness can augment self-awareness that, in-turn, is understood to improve ability to identify and label negative mood states and thinking patterns (Gillespie et al., 2012). This proposed mechanism relates closely to the ‘perceptual shift’ referred to above because increased accuracy in labelling mental states arguably makes it easier to objectify and observe them.
8. ***Addiction Substitution***: It has recently been proposed that the peaceful and blissful states associated with mindfulness can be used as a substitute for the ‘buzz’ or ‘high’ experienced by individuals with addiction problems (Shonin et al., 2014a). In essence, this mechanism of action involves substituting a ‘negative addiction’ (e.g., to drugs, alcohol, gambling, etc.) with a ‘positive addiction’ (i.e., to meditation/mindfulness). According to Van Gordon et al. (2016c), while individuals should eventually be encouraged to relinquish any dependency on meditation, it can be a useful technique in the early stages of addiction treatment.
9. ***Urge Surfing***: A further proposed mechanism of action relating to the use of mindfulness in addiction treatment contexts is that of ‘urge surfing’ (Appel & Kim-Appel, 2009). Urge surfing refers to the process of an individual observing and not reacting to mental urges. In other words, they ‘surf the urge’ and are thus better able to regulate addiction-related craving.
10. ***Letting Go***: By mindfully observing the arising and dissolution of cognitive, affective, and sensory processes, it is asserted that mindfulness practitioners cultivate a better understanding of the transient nature of existence (Van Gordon et al., 2016d). This helps to reduce attachment to (amongst other things) objects, people, situations, and concepts, and to encourage the ‘letting-go’ of negative affective states before they dominate thinking patterns and behaviour (Van Gordon et al., 2016e). Unlike Western psychology that generally deems attachment (e.g., in relationship contexts) to exert a protective influence over psychopathology, Buddhism asserts that attachment is an undesirable quality that leads to the reification of the ego-self (Sahdra et al., 2010). The Buddhist conceptualisation of attachment has been defined as “*the over-allocation of cognitive and emotional resources towards a particular object*, *construct*, *or idea to the extent that the object is assigned an attractive quality that is unrealistic and that exceeds its intrinsic worth*” (Shonin et al., 2014b, p. 126). Increased ability to let go of attachments is positively correlated with acceptance, non-reactivity, self-compassion, subjective wellbeing, and eudemonic wellbeing (Sahdra et al., 2010).

Various evidence-based mechanisms have been proposed to explicate the mechanistic pathways by which mindfulness improves mental health. Consistent with the biopsychosocial model of mental illness, these proposals suggest that mindfulness targets biological, psychological, and social (as well as spiritual) psychopathology determinants. It is unrealistic within this short paper to discuss all mechanisms of action that have been proposed to account for the treatment effectiveness of mindfulness. Furthermore, in the current paper, we specifically sought to introduce key emerging evidence-based proposals as well as those that are more empirically and theoretically established. Mindfulness undoubtedly exerts a therapeutic effect by exploiting multiple mechanistic pathways that are likely to vary according to factors such as (i) the type of MBI that is administered (e.g., first- or second-generation interventions), (ii) the specific clinical disorder that is being targeted, (iii) the educational, social, and spiritual history of the participant, and (iv) the extent to which the mindfulness instructor truly embodies the principles of mindful living. It is hoped that the mechanisms of mindfulness discussed in this paper will contribute to the formulation of a more complete picture that can be tested and expanded upon during future scientific enquiry.
